# Energy-dispersive X-ray micro Laue diffraction on a bent gold nanowire

**DOI:** 10.1107/S1600576720014855

**Published:** 2021-02-01

**Authors:** Ali AlHassan, A. Abboud, T. W. Cornelius, Z. Ren, O. Thomas, G. Richter, J.-S. Micha, S. Send, R. Hartmann, L. Strüder, U. Pietsch

**Affiliations:** a University of Siegen, Solid State Physics, Walter-Flex-Strasse 3, D-57072 Siegen, Germany; b Aix-Marseille Université, CNRS, Université de Toulon, IM2NP UMR 7334, 13397 Marseille, France; c Max Planck Institute for Intelligent Systems, Heisenbergstrasse 3, 70569 Stuttgart, Germany; d Université Grenoble Alpes, Institut de Recherches Interdisplinaires de Grenoble (IRIG) UMR SYMMES CNRS-CEA, and CRG-IF BM32 beamline at ESRF, Grenoble, France; e Université Grenoble Alpes, CEA/INAC, 17 rue des Martyrs, 38054 Grenoble, France; f PNSensor GmbH, Otto-Hahn-Ring 6, D-81739 München, Germany

**Keywords:** mechanical bending of Au nanowires, micro Laue diffraction, energy dispersive pnCCDs, atomic force microscopy, strain investigation

## Abstract

This article reports on energy-dispersive micro Laue (µLaue) diffraction of an individual gold nanowire that was mechanically deformed in three-point bending geometry using an atomic force microscope. The nanowire deformation was investigated by scanning the focused polychromatic X-ray beam along the nanowire and recording µLaue diffraction patterns using an energy-sensitive pnCCD detector that permits measurement of the angular positions of the Laue spots and the energies of the diffracted X-rays simultaneously. The plastic deformation of the nanowire was shown by a bending of up to 3.0 ± 0.1°, a torsion of up to 0.3 ± 0.1° and a maximum deformation depth of 80 ± 5 nm close to the position where the mechanical load was applied. In addition, extended Laue spots in the vicinity of one of the clamping points indicated the storage of geometrically necessary dislocations with a density of 7.5 × 10^13^ m^−2^. While µLaue diffraction with a non-energy-sensitive detector only gives access to the deviatoric strain, the energy sensitivity of the employed pnCCD offers absolute strain measurements with a resolution of 1%. Here, the residual strain after complete unloading of the nanowire amounted to maximum tensile and compressive strains of the order of +1.2 and −3%, which is comparable to the actual resolution limit. The combination of white-beam µLaue diffraction using an energy-sensitive pixel detector with nano-mechanical testing opens up new possibilities for the study of mechanical behavior at the nanoscale.

## Abstract 2

This article reports on energy-dispersive micro Laue (µLaue) diffraction of an individual gold nanowire that was mechanically deformed in three-point bending geometry using an atomic force microscope. The nanowire deformation was investigated by scanning the focused polychromatic X-ray beam along the nanowire and recording µLaue diffraction patterns using an energy-sensitive pnCCD detector that permits measurement of the angular positions of the Laue spots and the energies of the diffracted X-rays simultaneously.

## 1. Introduction   

Mechanical behavior at the nanoscale has attracted enormous attention in recent years. After the pioneering work of Uchic and co-workers on the microcompression of focused ion beam milled metal micropillars, which revealed increasing yield strength with decreasing feature size (Uchic *et al.*, 2004 ▸, 2009 ▸; Uchic & Dimiduk, 2005 ▸), studies on sub-micrometre defect-scarce specimens became the focus of researchers (Chen *et al.*, 2015 ▸; Richter *et al.*, 2009 ▸). This work showed ultra-high yield strength reaching the theoretical limit of the respective material. While early work concentrated on the *ex situ* characterization of the mechanically tested microstructures, *in situ* experimental setups have been developed in the past few years, allowing for the evolution of elastic and plastic deformation to be followed during mechanical loading. Among others, *in situ* transmission electron microscopy (TEM) measurements on gold nanowires that were cyclically deformed by compression and tension revealed twinning–detwinning which would not be detected by *ex situ* experiments (Lee *et al.*, 2014 ▸). However, high-resolution TEM, for example, is limited to electron-transparent samples which are less than 50–100 nm in thickness depending on the material.

Supported by suitable detection systems, the development of X-ray optics at third- and fourth-generation synchrotron radiation sources with high-brightness nano-focused X-ray beams (Johansson *et al.*, 2013 ▸; Martínez-Criado *et al.*, 2016 ▸; Leake *et al.*, 2019 ▸) has made it possible to probe the structural parameters, crystal phases and strain distribution of single nanostructures by means of X-ray-diffraction-based methods (Stankevič *et al.*, 2015 ▸; Cornelius & Thomas, 2018 ▸; Hill *et al.*, 2018 ▸; Lähnemann *et al.*, 2019 ▸). For example, high-resolution X-ray diffraction using a monochromatic beam focused to a diameter of a few hundred nanometres is commonly used to investigate the structural parameters of individual nanowires (Keplinger *et al.*, 2015 ▸; Stanchu *et al.*, 2015 ▸; Wallentin *et al.*, 2017 ▸). A monochromatic X-ray beam permits the inspection of specific individual Bragg reflections separately with high spatial resolution in reciprocal space, but this provides information about the strain profile only along the respective measured orientation (AlHassan *et al.*, 2018 ▸). This, in turn, requires precise and rather complicated diffraction geometries if several Bragg reflections need to be measured. Alternatively, X-ray diffraction using a polychromatic beam facilitates the measurement of multiple Bragg reflections at the same time without any movement or rotation of the goniometer. However, white-beam Laue diffraction does not give access to the full strain tensor, only to the deviatoric part of it owing to the lack of knowledge about the energy of the diffracted X-rays. By combining white-beam Laue diffraction with a two-dimensional energy-dispersive detector (pnCCD), the energy and angular position of each Laue spot can be directly accessed with a resolution of 1% and 0.06°, respectively (Send *et al.*, 2012 ▸; Abboud *et al.*, 2014 ▸). Altogether, the use of a pnCCD and a white X-ray beam provides detailed information about the lattice deformation which cannot be achieved using conventional non-energy-dispersive 2D detectors. While the combination of µLaue diffraction and an energy-dispersive pnCCD has been mainly used to investigate strain in microstructures and polycrystalline materials, we present here the first energy-dispersive µLaue diffraction study on a single Au nanowire that was priorly deformed mechanically in a three-point bending configuration using a custom-built atomic force microscope (AFM), demonstrating the feasibility of this technique for the analysis of nano­structures.

The combination of atomic force microscopy and simultaneous µLaue diffraction performed on an individual self-suspended Au nanowire was previously demonstrated by Leclere *et al.* (2015 ▸). The experiment was carried out at beamline BM32 at the ESRF using a white beam with an energy of 5–25 keV and a non-energy-sensitive MarCCD165 detector (now Rayonix). Laue patterns were acquired at a fixed position along the nanowire growth axis, 1.8 µm away from the position where different magnitudes of mechanical force were applied by the AFM, revealing the displacement of the Au Laue diffraction spots on the detector as a function of AFM loading. This was further improved by scanning the X-ray beam along the whole nanowire using the pair of Kirkpatrick–Baez (KB) mirrors employed for focusing the X-ray beam instead of translating the sample across the beam (Leclere *et al.*, 2016 ▸). Furthermore, the authors studied the nanowire plasticity (Ren *et al.*, 2018 ▸). The key difference in the present work is the use of a pnCCD as a detection tool, taking advantage of the extra energy dimension it provides to quantify the strain in the nanowire. This was not accessible in the above-mentioned experiments. As a result, it is possible to investigate the impact of mechanical force on the structural properties of an individual Au nanowire. By scanning the focused X-ray beam along the nanowire in steps of 1 µm, the response of four selected lattice planes and thereby the evolution of elastic and plastic strain after mechanical unloading is revealed as a function of the spatial position.

## 2. Sample description and experimental setup   

**Figure 1:**
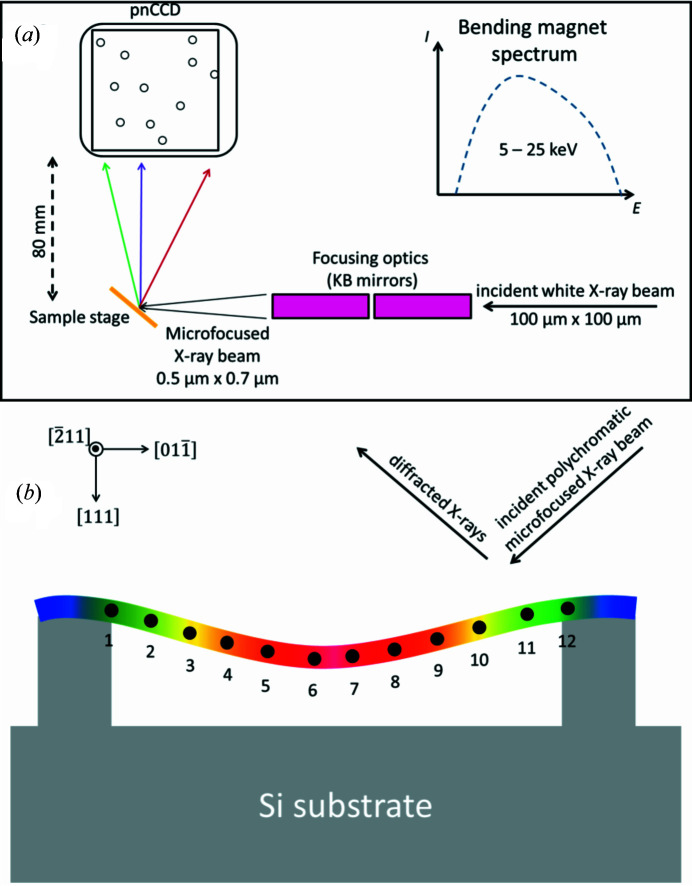
Experimental setup and scanning geometry. (*a*) The energy spectrum of the white X-ray beam ranges from 5 to 25 keV. The beam was focused down to 0.5 × 0.7 µm by a pair of Kirkpatrick–Baez mirrors. The sample-to-detector distance was about 8 cm and the scattering intensity was collected at an angle of about 90°. (*b*) The nanowire is self-suspended across an Si micro-trench and lies on one of its 〈111〉 side facets with its growth direction along [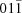]. The mechanically deformed nanowire is scanned by a nano-focused white X-ray beam at 12 different positions along its growth axis, marked in black and numbered from 1 to 12.

Single-crystalline gold nanowires with diameters of a few hundred nanometres and lengths of a few tens of micrometres were grown by physical vapor deposition on carbon-coated tungsten substrates under ultra-high-vacuum conditions at elevated temperatures (∼1020 K) (Richter *et al.*, 2009 ▸). The as-grown Au nanowires were transferred onto an Si substrate patterned with 10 µm wide, 1.5 µm deep and 1 mm long micro-trenches by scratching the growth substrate against it. Some of the randomly deposited nanowires crossed the Si micro-trenches forming self-suspended nanobridges (Ren *et al.*, 2018 ▸). To avoid any sliding during the subsequent bending experiments, the suspended nanowires were securely attached at the Si ridges by electron-beam-induced deposition of carbon from the residual gas in a scanning electron microscopy chamber [see Fig. 1 ▸(*b*)]. The investigated nanowires are about 20 µm in length and 350 nm in diameter. Their growth direction is along [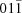] and they lie on the micro-trenches with one of their 〈111〉 side facets [Fig. 1 ▸(*b*)]. A selected Au nanowire was mechanically deformed in three-point bending configuration using the custom-built AFM ‘SFINX’ (Ren *et al.*, 2014 ▸), which was designed for *in situ* nano-mechanical testing at synchrotron beamlines (Leclere *et al.*, 2015 ▸; Ren *et al.*, 2018 ▸). For nano-mechanical loading of the nanowire, the SFINX Si tip was lowered with a constant speed using the piezoelectric stage carrying the SFINX head. Once the Si tip was in contact with the nanowire, it was moved further by 600 nm (corresponding to a force of about 1 µN as demonstrated by finite element method simulations (Ren *et al.*, 2018 ▸), thus pushing against the nanowire and deflecting it.

After complete unloading of the nanowire by retracting the SFINX Si tip, µLaue diffraction was performed at beamline BM32 at the ESRF using a polychromatic beam with an energy range of 5–25 keV. The incident beam was focused down to a spot size of 0.5 × 0.7 µm on the sample surface, which is inclined by 40° with respect to the incident polychromatic X-ray beam, using a pair of KB mirrors [see Fig. 1 ▸(*a*)]. At first, a large-area non-energy-dispersive MarCCD with 2048 × 2048 pixels of pixel size 80 × 80 µm installed at 90° and at a distance of 70 mm from the sample position was used for collecting µLaue diffraction patterns. Thanks to the large solid angle covered by this detector, a multitude of Laue spots originating both from the Au nanowire and from the Si substrate were measured (see Fig. S1 in the supplementary material). The µLaue diffraction patterns were indexed using the *LaueTools* software (Micha, 2014 ▸).

This detector was then replaced by an energy-dispersive pnCCD, defined as a pn-junction charge coupled device, which was used for detecting the intensity diffracted by the deformed wire. The detection area of the pnCCD is composed of 384 × 384 pixels with a pixel size of 75 × 75 µm. The smaller detection area compared with the MarCCD reduces the number of detected Laue spots, while each pixel serves as an individual energy-selective point detector with an energy resolution Δ*E*/*E* of about 1%. The sensitive area of the pnCCD, which measures 28.8 × 28.8 mm, is composed of 450 µm thick n-doped Si. The sensitive area is subdivided into two parts: (1) an image area of 384 × 384 pixels with 75 × 75 µm pixel size, and (2) a frame store area of 384 × 384 pixels with 75 × 51 µm pixel size and a typical readout frequency of about 100 Hz. The sample-to-detector distance was about 8 cm and the scattered intensity was collected at an angle of about 90° with respect to the incident X-ray beam. An Al absorber was installed upstream of the sample in order to reduce the incident flux.

## 3. Results   

**Figure 2:**
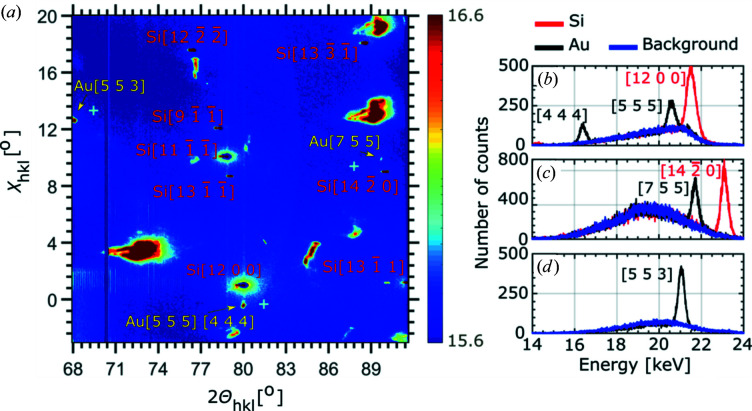
(*a*) Full detector frame (384 × 384 pixels) recorded for position 9 along the nanowire growth axis, demonstrated in Fig. 1 ▸. Spatially separated from the Si Laue reflections which are indexed in red, four neighboring Au reflections (553, 755, 444 and 555) are visible. (*b*)–(*d*) Energy spectra extracted for the four Au and Si 12 0 0 and 14 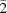 0 reflections are plotted in the range of 14 to 24 keV. Blue, black and red line profiles represent the energies of the background, Au and Si, respectively. The positions at which the background was extracted are indicated by light-blue crosses in (*a*).

As illustrated in Fig. 1 ▸, the nanowire was measured at 12 different positions along its growth axis in steps of 1 µm. The µLaue patterns at positions 1 and 12 were recorded at the clamping points, and scan number 6 corresponds to the loading position. The full detector image displayed in Fig. 2 ▸(*a*) was taken at measurement position 9. It includes sharp diffraction signals that are attributed to the Au nanowire and the Si substrate, highly intense and deformed spots, and signals with sub-peaks that were not observed using the MarCCD. The last may originate from the detector housing and are therefore not taken into account in our calculations.

Considering the energies of the respective Laue spots and their angular separation, in addition to comparing the pnCCD diffraction pattern with the indexed MarCCD Laue diffraction pattern (see Fig. S1 in the supplementary material), eight Si and three Au Laue spots are indexed with their respective Miller indices *hkl* (Fig. S2 in the supplementary material). Following the indexing process, the absolute angular position of each pixel of the pnCCD diffraction patterns was calibrated. The two Au reflections at the right and left edges of the detector image shown in Fig. 2 ▸(*a*) are indexed as 755 and 553, respectively. Close to the Si 12 0 0 Laue spot, a third Au Laue spot exists which actually consists of two diffraction signals from the same family of lattice planes (harmonics) [Fig. 2 ▸(*a*)]. In the case of Laue diffraction, harmonics overlap on the detector and cannot be distinguished by conventional non-energy-dispersive detectors. The pnCCD, on the other hand, allows for measuring two distinct energies for the Au Laue spot [as shown in Fig. 2 ▸(*b*) and discussed below], which facilitates distinguishing diffraction signals of the same family of lattice planes {*hkl*}.

The energy spectrum of each pixel of the pnCCD was calibrated using the literature values of the fluorescence energies of the respective materials (see Fig. S3 in the supplementary material) (Thompson *et al.*, 2009 ▸). The calibrated energy spectra extracted at the Si 12 0 0 and the nearby Au Laue spot, the Au 755 and Si 14 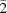 0 spots, and the Au 553 Laue spot are presented in Figs. 2 ▸(*b*), 2 ▸(*c*) and 2 ▸(*d*), respectively. The energy spectra of the Si Laue spots, the Au Laue spots and the background spectrum extracted far out of the Bragg reflections [light-blue crosses in Fig. 2 ▸(*a*)] are colored in red, black and blue, respectively. The Au Laue spot close to the Si 12 0 0 diffraction signal exhibits two peaks in energy at ∼16.5 and ∼20.75 keV [Fig. 2 ▸(*b*)], corresponding to the Au 444 and Au 555 Laue spots. The pnCCD was installed at an angle of ∼90° with respect to the incident X-ray beam. Thus, mostly high-energy reflections are accessible. In addition, an Al absorber mounted upstream from the sample as well as 1–2 m of air significantly absorbed low-energy photons and soft X-rays. Therefore, lower-index symmetric Laue reflections related to lower-energy photons were not detected.

**Figure 3:**
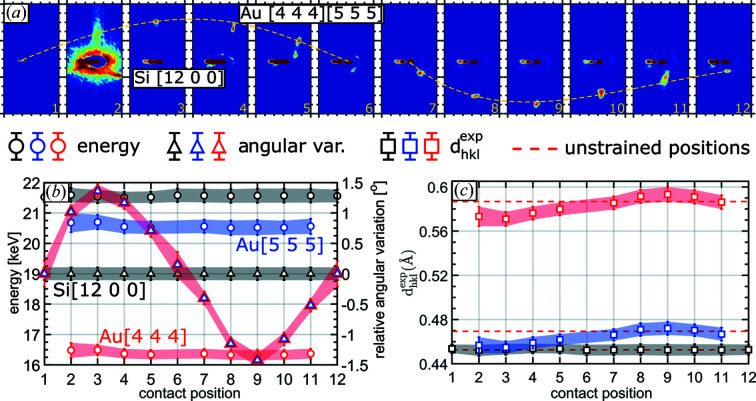
(*a*) Evolution of the Au 444 and 555 Laue spots as a function of the measurement position along the nanowire growth axis, denoted by 1–12 in Fig. 1 ▸. The movement of the Au Laue spots is underlined by a yellow dashed line. (*b*) Energy, angular and (*c*) *d*-value variation of Au 444 and 555 and Si 12 0 0 as a function of the beam position along the nanowire growth axis. Red dashed lines in (*c*) indicate the unstrained positions of Au 444 and 555 and Si 12 0 0 from top to bottom, respectively.

The evolution of the Au 444 and 555 Laue spots along the nanowire is illustrated in Fig. 3 ▸(*a*), showing a sinusoidal-like movement comparing the 12 diffraction patterns. In order to obtain the angular variation of the Laue spots, which reflect the angular tilt of their respective lattice planes, each peak was fitted by a single Gaussian. As demonstrated in Fig. 3 ▸(*b*), the angular variation of the two Laue spots ranges from −1.4 to +1.4°. The spatial position of the Si 12 0 0 Laue spot and therefore the crystalline orientation remain unchanged. Note that the diffraction pattern at position 2 is plotted with a different color scale to increase the visibility of the Au 444 and 555 Laue spots. The angular uncertainty is approximated here to be 2 pixels in each direction, corresponding to ±0.105°.

Note that the Au 444 and Au 555 Laue spots overlap with the Si 12 0 0 Laue spot in the first and last measurements as well as at measurement position 6. The relative angle of the Au Laue spots at measurement position 6 was chosen considering half the angular difference between positions 5 and 7, whereas the angle at measurement positions 1 and 12 was chosen to be 0 with an error of 4 pixels in each direction (corresponding to ±0.21°) assuming that the nanowire was well clamped to the Si ridges of the substrate.

Knowing both the energy and the Bragg angle of each Laue spot, the interplanar spacing, 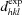, of the corresponding reflection in reciprocal space or set of lattice planes in real space can be calculated using Bragg’s law:
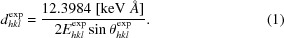



In a cubic crystal system, the square of the measured *d* value and the square of the lattice parameter, *a*, are connected by the sum of squares of Miller indices *h* ^2^ + *k* ^2^ + *l* ^2^. The variation in the interplanar spacing 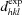 for Au 444, Au 555 and Si 12 0 0 is presented in Fig. 3 ▸(*c*), where the respective theoretical *d* values are represented by dashed red lines. Owing to the overlapping of the Au Laue spot with the Si Laue spot at measurement position 6 and the low intensity in the first and last measurement positions, the energies for these data points are missing for the Au reflection. As expected, Si 12 0 0 shows no energy variation for the 12 measurements carried out along the nanowire growth axis. The *d* values of the Au (444) and Au (555) lattice planes vary by about 0.01 A˙. This variation is within the error bars of the measurements, comprising the uncertainties in the determination both of the energy (Δ*E*/*E* ≃ 1%) and of the angle (∼0.1°). The angular variation, the energy and the inferred *d* spacing of the Au 553 Laue spot and of the Au 755 and Si 14 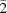 0 Laue spots are displayed in Figs. S4 and S5 in the supplementary material, respectively.

The Au 444 and Au 555 Laue spots exhibit a well defined circular shape along the nanowire except at measurement positions 10 and 11. While a well defined round diffraction signal indicates a perfect crystal, the spatial extension (streaking) of the diffraction spot implies the presence either of defects or of a strain gradient (Shokr *et al.*, 2020 ▸). Within the resolution limit, the energy of the diffracted X-rays does not vary along the two diffraction spots (as illustrated in Figs. S6 and S7 in the supplementary material), which suggests that the lattice spacing does not vary and, thus, the elongation of the Laue spot does not originate from a strain gradient but indicates bending of lattice planes caused by dislocations stored in the probed volume (which is discussed below).

**Figure 4:**
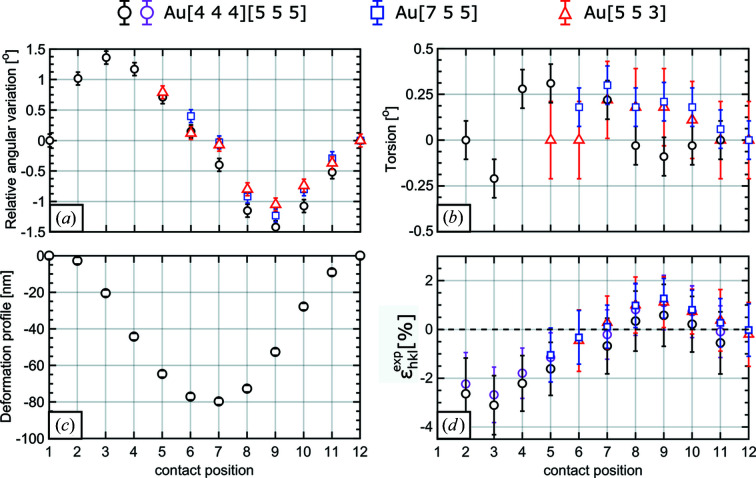
(*a*), (*b*) Angular variation (bending angle) and torsion of the 444, 555, 755 and 553 lattice planes of the bent Au nanowire. (*c*) The deformation (bending) profile of the Au nanowire. (*d*) The strain variation along the length of the nanowire axis for the respective lattice planes.

From the angular variations of the three Au Laue spots, the bending and torsion of the Au nanowire were inferred, being displayed in Figs. 4 ▸(*a*) and 4 ▸(*b*), respectively. Because the Au 553 and Au 755 Laue spots were either outside of the camera or overlapping with another signal on the detector during the first few measurements, these data points are missing for these two diffraction signals. The bending angle of the nanowire shows a sinusoidal-like behavior (similar to the variation of the Laue spots themselves on the detector), varying from −1.4 to +1.4°. However, the nanowire shows only a minor torsion of less than 0.3°, which is close to the actual angular uncertainty. The deformation profile of the nanowire inferred from the bending angles of the Au 444 and Au 555 lattice planes, which are parallel to the nanowire surface, shows a maximal deformation of 80 nm [Fig. 4 ▸(*c*)]. The measurement uncertainty is the same size as the symbols or smaller.

From the 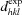 values presented in Fig. 3 ▸(*c*) and the literature values 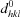 of the respective lattice planes, the strain along the nanowire was calculated using the following equation:
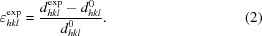



The strain values for all four Au lattice planes measured in this experiment show exactly the same behavior, as illustrated in Fig. 4 ▸(*d*). The maximum tensile and compressive strains are of the order of +1.2 and −3% with an error of ±1.5%. Considering that the determination of the variation of the *d* spacing was close to the actual resolution limit, the same is true for the absolute strain.

## 4. Discussion   

White-beam Laue diffraction is highly sensitive to the orientation of lattice planes; however, it does not provide access to the absolute strain but rather to the deviatoric strain inside a structure. This deficiency comes from the fact that the energy of the diffracted X-rays is unknown. Here, we demonstrated the use of an energy-dispersive pnCCD detector on a plastically deformed Au nanowire. The reduced size of the active area of the pnCCD compared with standard non-energy-dispersive detectors limits the number of Laue spots that can be measured in one frame, and thus *UB* orientation matrices cannot be obtained. As demonstrated in previous work (Leclere *et al.*, 2016 ▸), the crystalline orientation can be monitored by the displacement of the Laue spots on the camera, evidencing here bending angles that range from −1.4 ± 0.1° to +1.4 ± 0.1°, torsions of up to 0.3 ± 0.1° and a maximum deformation depth of 80 ± 5 nm. Previous *in situ* three-point bending experiments have shown torsions of more than 9° that relaxed to less than 4° upon unloading (Ren *et al.*, 2018 ▸). The nanowire torsion was attributed to the misalignment of the AFM tip used for the mechanical loading with respect to the nanowire center by a few tens of nanometres. The fact that the nanowire torsion in the present work is practically zero indicates that the loading point was well centered on the nanowire top facet.

Considering that the nanowire was mechanically unloaded prior to the diffraction measurement, the bending, torsion and deformation profile indicate that the nanowire was deformed plastically. This assumption was confirmed by the extension of the Laue diffraction spots at one of the clamping positions, which was caused by defects that are stored in the probed volume. These findings are in agreement with previous results that showed the nucleation of dislocations in the vicinity of the clamping point where the stress is concentrated (Ren *et al.*, 2018 ▸, 2020 ▸). A distribution of geometrically necessary dislocations (GNDs) causes asymmetric broadening of Laue peaks (Barabash *et al.*, 2002 ▸). Comparing the direction of the broadening of the diffraction pattern allows for the determination of the active slip systems. The fact that the Laue spot in the present work is not only elongated along one direction but is also widened laterally indicates the activation of at least two slip systems or the presence of statistically stored dislocations. According to Nye (1953 ▸) the plastic deformation of a crystal by an angle Δθ is directly related to the density of stored GNDs:
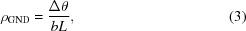
where *b* is the magnitude of the Burgers vector and *L* is the probed length (here, the focal size of the X-ray beam of 500 nm) along **b**.

Considering only the main elongations of the diffraction peaks for the measurement positions 10 and 11, which are 0.42 and 0.62°, respectively (this is much larger than the divergence of the incident X-ray beam of a few milliradians), results in dislocation densities of 5.1 and 7.5 × 10^13^ m^−2^. This compares with the minimum detectable dislocation density of 2.5 × 10^13^ m^−2^ along the rest of the nanowire, where well defined circular Laue diffraction spots were measured with an angular extension of 0.21° representing the lower-resolution limit of this technique.

The energy sensitivity of the pnCCD used in the current work allows for the differentiation of Laue spots originating from the same family of lattice planes {*hkl*} (harmonics; which are superimposed and thus cannot be distinguished using non-energy-dispersive detectors) and principally provides access to the absolute strain. Regarding the energy resolution of the detector, the measured strain values of the order of 1% are actually at the resolution limit. Considering that the nanowire was plastically deformed and mechanically unloaded prior to the measurement, the elastic strain is expected to be relaxed. The bending and secure clamping at both ends, however, may induce a tensile strain along the nanowire despite its plastic relaxation, which induces a compressive strain along the Au [111] direction by the Poisson effect. This Poisson-induced compressive strain is expected to be far below the detection limit of about 1%. Considering a similar Au nanowire bent elastically to bending angles of up to 3°, the maximum strain calculated by the finite element method amounted to only 0.15% (Leclere *et al.*, 2015 ▸).

The Fano limit of Si is FWHM = ∼130 eV at 8 keV, giving a resolution Δ*E*/*E* = 0.016. Monochromatic diffraction uses beams with an energy resolution of Δ*E*/*E* = 10^−4^, corresponding to a few eV. While the Fano limit amounts to >100 eV, the actual energy resolution determined from the energy distribution of a diffraction peak is of the order of a few eV. This makes the two techniques comparable, but only on a relative scale.

This experiment is the first demonstration of *ex situ* energy-dispersive µLaue diffraction on a plastically deformed nanostructure. While the present energy resolution of the employed pnCCD limited the measurement of the absolute strain in the Au nanowire, future energy-dispersive detectors with improved energy resolution will open up new possibilities for strain-resolved white-beam µLaue diffraction. Future pnCCDs with larger active areas will further allow for detecting a larger number of Laue spots, thus facilitating the determination of the *UB* orientation matrices and finally measurement of the complete strain tensor. In addition, while the exposure time in the present experiment was of the order of one hour per frame because of the long dead time of the detector, next-generation pnCCDs with shorter dead times will eventually facilitate their usage in *in situ* nano-mechanical experiments where stability issues are crucial.

## 5. Conclusion   

In conclusion, energy-dispersive µLaue diffraction was performed on a single gold nanowire. By means of an AFM tip, the Au nanowire was mechanically bent at its mid-section by a force of about 1 µN, resulting in a measured bending of up to 1.4 ± 0.1°, a torsion of up to 0.3 ± 0.1° and a maximum deformation depth of 80 ± 5 nm at the position where mechanical force was applied. By means of an energy-sensitive pnCCD as detection tool, the lattice deformation along the growth axis of the nanowire was determined. This was not possible before using conventional non-energy-sensitive detectors. However, the limited energy resolution of 1%, which results in a strain resolution of the same order, hampered the measurement of the lattice deformation which is expected to be of the order of a few tenths of a percent. While Laue spots of the same family of lattice planes spatially overlap, and thus cannot be distinguished using conventional detectors, the pnCCD facilitated the discrimination between the Au 444 and Au 555 Laue spots. In the present work, all measured (*hkl*) belong to the same zone in reciprocal space. As demonstrated by Abboud *et al.* (2017 ▸), the measurement of reflections belonging to different crystallographic zones provides access to all components of the strain tensor with rather high accuracy. Therefore the technique may serve as an effective tool to evaluate strain variations of nano-objects as a function of different kinds of deformation.

## Supplementary Material

Supporting information file. DOI: 10.1107/S1600576720014855/vh5131sup1.pdf
