# Supplementary material for: Energy-dispersive X-ray micro Laue diffraction on a bent gold nanowire
Source: J Appl Crystallogr. 2021 Feb 1;54(Pt 1):80–6. doi: 10.1107/S1600576720014855 (PMC7941300; doi:10.1107/S1600576720014855)
Supplement: Supplementary file 1 [file j-54-00080-sup1.pdf]

JOURNAL OF
APPLIED
CRYSTALLOGRAPHY

Volume 54 (2021)

Supporting information for article:

Energy dispersive X-ray micro Laue diffraction on a bent gold nanowire

Ali AlHassan, A. Abboud, T. W. Cornelius, Z. Ren, O. Thomas, G. Richter, J.-S. Micha, S. Send, R. Hartmann, L. Strüder and U. Pietsch

S1. Part I: MarCCD Laue pattern

Laue microdiffraction patterns were recorded using a large area MarCCD165 detector (now rayonix) with 2048 x 2048 pixels with a size of 80 µm which installed at a distance of 70 mm at an angle of 90°, thus covering a solid angle of about 100 °. A µLaue diffraction pattern recorded at the position of the nanowire is presented in Fig. S1 showing diffraction signals of both the Au nanowire and the Si substrate. The shadow on the right hand side is caused by the head of the AFM that was used to deform the Au nanowire prior to the installation of the energy-dispersive pnCCD detector. The µLaue diffraction patterns were indexed using the LaueTools software.

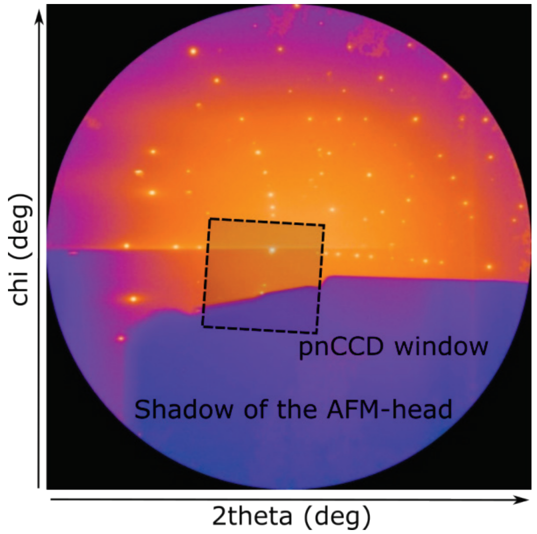

Figure S1 2048 x 2048 pixels µLaue diffraction pattern recorded using a MarCCD165 detector (now rayonix). The pnCCD detection window is overlayed with the MarCCD detector and indicated by a dashed square. The bottom side of the pattern is shaded by the AFM head.

The simulated µLaue diffraction patterns for the Au nanowire and the Si substrate, using their respective UB orientation matrices, is presented in Fig. S2. The area in reciprocal space probed by the pnCCD is highlighted by a dashed square and the Au and Si Laue spots actually recorded by the energy dispersive detector are marked by red circles and blue squares, respectively. An enlarged view of the detected area in reciprocal space is displayed in Fig. 1(c).

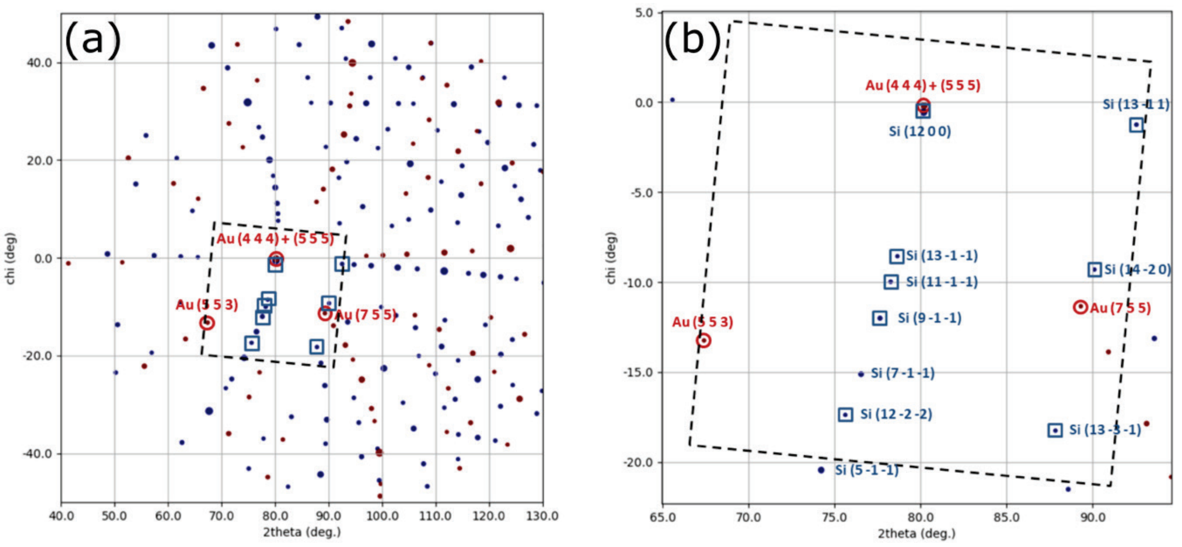

Figure S2 (a) Simulated μLaue diffraction pattern for the Au nanowire and the Si substrate. The dashed box represents the detection area of the pnCCD. (b) Higher magnification of the area indicated by a dashed square in (a).

S2. Part II: Energy calibration

As the incident beam penetrates through the nanowire and underlying substrate, fluorescence peaks that originate from the detector housing and experimental setup, i.e. Fe and Ni, may appear in the energy spectrum. An energy spectrum integrated over an area of 100 x 100 pixels is extracted from the diffraction pattern recorded at measurement position 9 along the nanowire length and is demonstrated in Fig. S3. Energy peaks named from 1-10 are listed and explained in Table 1. Before calibration, the spectrum is represented by channel numbers. The channel number is then translated to energy by setting one of the fluorescence peaks, e.g. Fe K_α to its theoretical value of 6.403 keV. The factor by which the channel numbers are multiplied is called the “gain factor”. The calibrated energy values are demonstrated in the lower x-axis of Fig. S3.

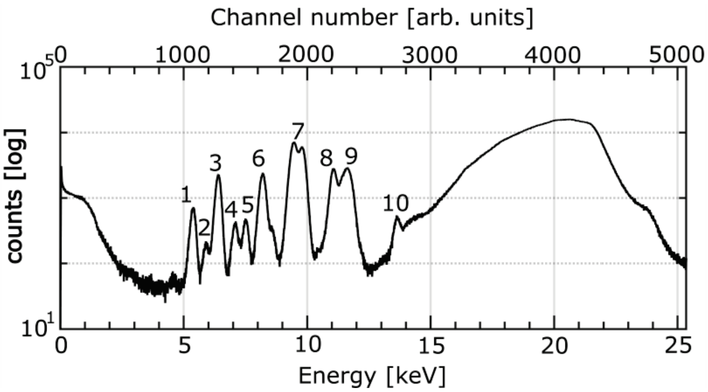

Figure S3 Energy spectrum extracted at measurement position 9 along the nanowire length before (a) and after (b) calibration. Peak 1-10 are explained in the table below.

Table S1 Fluorescence peaks with their energies and types, named 1-10 in Fig. S3(a).

peaks	1	2	3	4	5	6
Energy (keV)	5.414	5.946	6.403	7.057	7.477	8.047 + 8.264
type	Cr k_{α}	Cr k_{β}	Fe k_{α}	Fe k_{β}	Ni k_{α}	Cu k_{α} + Ni k_{β}
peaks	7	8	9	10		
Energy (keV)	9.441 + 9.711	11.069	11.439	13.379	25 keV	>25 keV
type	Pt L_{α} + Au k_{α}	Pt L_{β}	Au L_{β}	Au L_{γ}	end of spectrum	pile up

S3. Part III: Analyzed Au Laue spots

Four Au Laue spots are recorded in the detector frame displayed in Fig. 2(a). Au (4 4 4) and (5 5 5) overlap at the same spatial position and are discussed in the main text. Au (5 5 3) and (7 5 5) are appear at the left and right edges of the detector window. The angular variation, energies and spacing of the corresponding Laue spots, and thus lattice planes are shown in Figs. S4 and S5. The mean strain along the nanowire full length is calculated to be ≈ –1.4% ± 1.0% for Au (5 5 3) and –2.1% ± 1.0% for Au (7 5 5). For Au (5 5 3), the maximum compression of ≈ –3.1% ± 1.1% takes place at contact position 5 whereas the minimum compression of ≈ –0.6 ± 0.9% is at contact position 9. The angular displacement is ≈ 1.6° comparing contact positions 5 and 9.

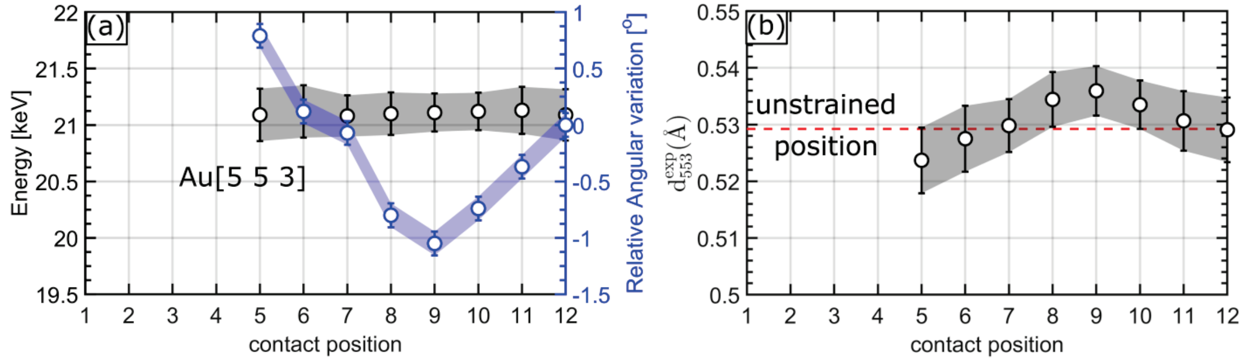

Figure S4 (a) Energy, angular and (b) d-value variation of Au (5 5 3) as a function of the beam position along the nanowire growth axis. The angular variation is represented in blue color. The red dashed line in (b) indicates the unstrained d-value of Au (5 5 3).

For Au (7 5 5), the maximum compression of ≈ –2.4 ± 1.0% takes place at contact position 6 whereas the minimum tensile of ≈ –1.6 ± 0.8% is at contact position 9. The angular displacement is ≈ 1.1° comparing contact positions 9 and 12.

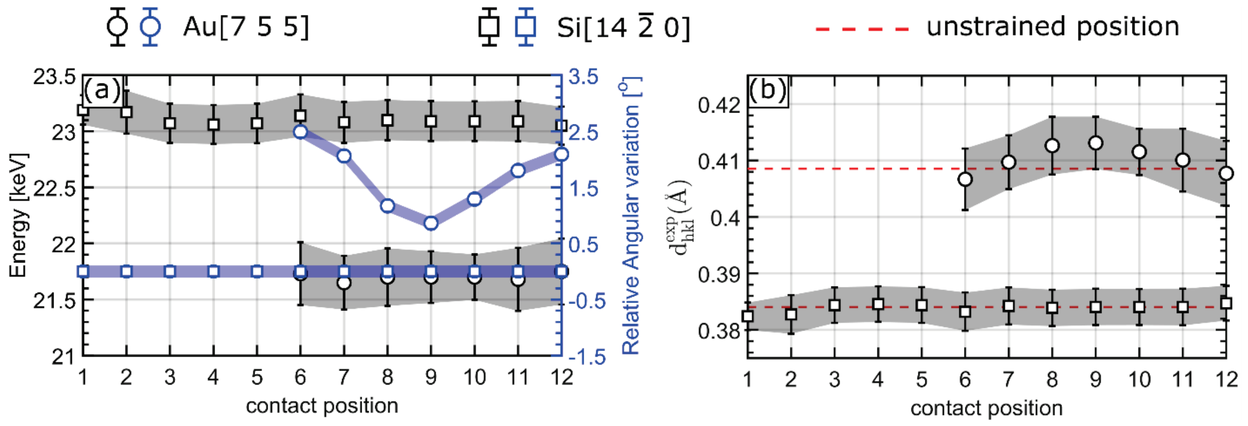

Figure S5 (a) Energy, angular and (b) d-value variation of Au (7 5 5) and Si (14 $\bar{2}$ 0) and (c) as a function of the beam position along the nanowire growth axis. The angular variation is represented in blue color.

S4. Part IV: Elongation of Au (4 4 4) and (5 5 5)

As mentioned in the main text, the Au (4 4 4) and (5 5 5) Laue spots are elongated at measurement positions 10 and 11 along the nanowire growth axis. In this part, we study the energy variation of the Laue spots along their streaking direction. This is achieved by extracting energy spectra in areas of 1 pixel x 1 pixel at different positions along their elongation profiles. The results for

measurement positions 10 and 11 are demonstrated in Figs. S6 and S7, respectively i.e. no energy variation is observed along the elongation direction of Au (4 4 4) and (5 5 5). The elongation of the Laue spots is approximated by 0.42° and 0.62° at measurement positions 10 and 11, respectively.

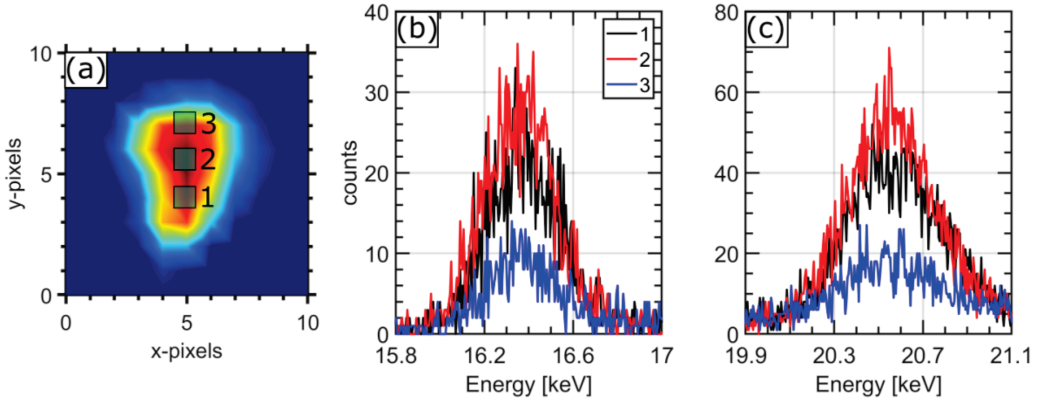

Figure S6: (a) Au (4 4 4) and (5 5 5) at contact position 10. (b) and (c) show the energy peaks of Au (4 4 4) and (5 5 5). The positions at which the energy spectra were extracted are indicated by square boxes in (a). Each box is 1 pixel x 1 pixel in size.

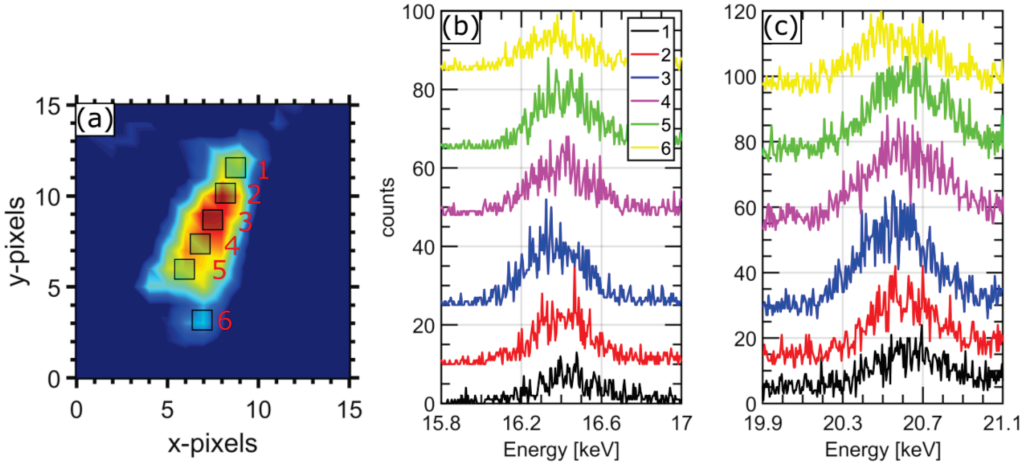

Figure S6 Au (4 4 4) and (5 5 5) at contact position 11. (b) and (c) show the energy peaks of Au (4 4 4) and (5 5 5). The positions at which the energy spectra were extracted are indicated by square boxes in (a). Each box is 1 pixel x 1 pixel in size. The spectra are plotted with a vertical offset for better clarity.
